# Colyophilized Sugar–Polymer Dispersions for Enhanced Processing and Storage Stability

**DOI:** 10.1021/acs.molpharmaceut.4c00187

**Published:** 2024-05-17

**Authors:** Claudia Giannachi, Evin Allen, Gráinne Egan, Sonja Vucen, Abina Crean

**Affiliations:** † SSPC, the SFI Research Centre for Pharmaceuticals, School of Pharmacy, University College Cork, Cork T12 YT20, Ireland; ‡ School of Pharmacy, University College Cork, Cork T12 YT20, Ireland

**Keywords:** lyophilization, glass transition temperature, hydrogen bonding, amorphous solid dispersions, humidity, stability

## Abstract

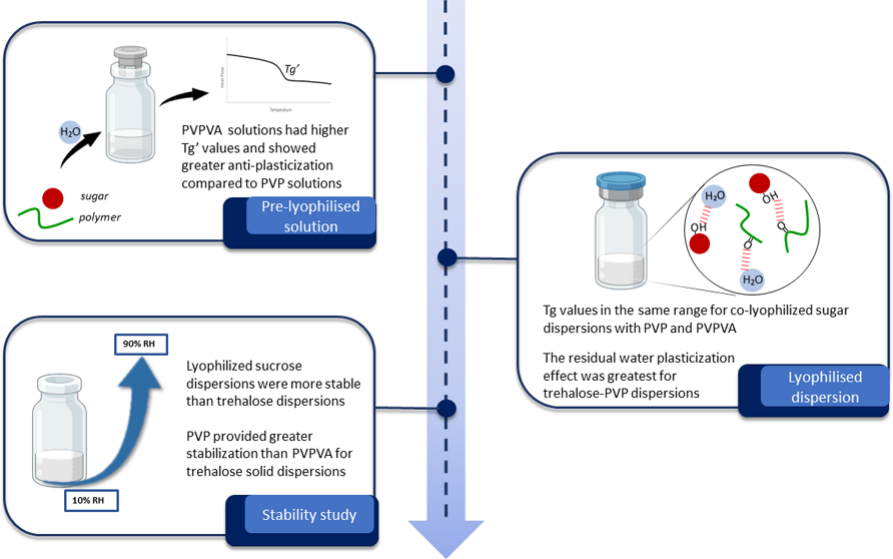

Sucrose and trehalose pharmaceutical excipients are employed to stabilize protein therapeutics in a dried state. The mechanism of therapeutic protein stabilization is dependent on the sugars being present in an amorphous solid-state. Colyophilization of sugars with high glass transition polymers, polyvinylpyrrolidone (PVP), and poly(vinylpyrrolidone vinyl acetate) (PVPVA), enhances amorphous sugar stability. This study investigates the stability of colyophilized sugar–polymer systems in the frozen solution state, dried state postlyophilization, and upon exposure to elevated humidity. Binary systems of sucrose or trehalose with PVP or PVPVA were lyophilized with sugar/polymer ratios ranging from 2:8 to 8:2. Frozen sugar–PVPVA solutions exhibited a higher glass transition temperature of the maximally freeze-concentrated amorphous phase (*T*_g_’) compared to sugar–PVP solutions, despite the glass transition temperature (*T*_g_) of PVPVA being lower than PVP. *T*_g_ values of all colyophilized systems were in a similar temperature range irrespective of polymer type. Greater hydrogen bonding between sugars and PVP and the lower hygroscopicity of PVPVA influenced polymer antiplasticization effects and the plasticization effects of residual water. Plasticization due to water sorption was investigated in a dynamic vapor sorption humidity ramping experiment. Lyophilized sucrose systems exhibited increased amorphous stability compared to trehalose upon exposure to the humidity. Recrystallization of trehalose was observed and stabilized by polymer addition. Lower concentrations of PVP inhibited trehalose recrystallization compared to PVPVA. These stabilizing effects were attributed to the increased hydrogen bonding between trehalose and PVP compared to trehalose and PVPVA. Overall, the study demonstrated how differences in polymer hygroscopicity and hydrogen bonding with sugars influence the stability of colyophilized amorphous dispersions. These insights into excipient solid-state stability are relevant to the development of stabilized biopharmaceutical solid-state formulations.

## 1 Introduction

Lyophilization is a commonly employed processing approach to provide protein therapeutics with adequate shelf life/stability for commercialization.^1−3^ Lyophilization consists of gradual water removal through sublimation of ice from the frozen state followed by bound moisture desorption under vacuum.^4^ Water removal reduces the molecular mobility and allows therapeutic protein formulations to maintain stability during drying and storage. Despite being a conservative drying process, proteins are exposed to freezing and drying stresses during lyophilization that can destabilize the protein structure via interfacial adsorption and cryo-concentration.^5,6^ A proven strategy to overcome this destabilization is to dry proteins in the presence of stabilizers, which are commonly nonreducing sugars, e.g., sucrose and trehalose.^7,8^

Two hypotheses have been proposed to explain how sugars stabilize proteins in the dried state: the “water replacement hypothesis” which is a thermodynamic stabilization mechanism and “vitrification hypothesis” which is a kinetic stabilization mechanism.^9−12^ The “water replacement hypothesis” proposes that sugar hydroxyl groups form hydrogen bonds with the protein surface and “replace” the hydrogen bond interaction with water lost during drying.^4,13−16^ The “vitrification hypothesis” proposes the protein is immobilized in a rigid and amorphous glassy sugar matrix in which chemical and conformational degradation that requires mobility is prevented or minimized.^4,10,15−18^ Both hypotheses require the stabilizing sugar to be in a glassy amorphous state.^19^ However, amorphous sucrose and trehalose glasses are thermodynamically unstable and can transform to more stable crystalline forms above their glass transition temperature (*T*_g_). Amorphous sugar *T*_g_ can be reduced due to the plasticizing effects of residual and environmental moisture, requiring careful consideration of process and formulation choices.^20,21^

A common strategy to stabilize amorphous small organic therapeutics for oral administration is their coformulation with a higher *T*_g_ polymer creating a drug-polymer solid dispersion. Within these amorphous solid systems, the drug is stabilized in an amorphous state due to an increase in *T*_g_ and intermolecular bonding between the drug and polymer.^22^ This approach has been investigated to a more limited extent to stabilize sucrose and trehalose in an amorphous state employing pharmaceutical-approved polymers, polyvinylpyrrolidone (PVP), and polyvinylpyrrolidone-*co*-vinyl acetate (PVPVA).^20,23−25^

The *T*_g_ transitions and intermolecular interactions of colyophilized sugar–PVP and sugar–PVPVA dispersions have been studied and are relatively well understood.^20,23,26,27^ In all cases, the combination of the polymer with sugar increased the system’s *T*_g_ relative to the *T*_g_ of the amorphous sugar alone. A greater increase in the *T*_g_ of the PVP systems was noted compared to that of the PVPVA systems; a trend that is in line with the difference in *T*_g_ of the two polymers.^26^ Employing Fourier-transform infrared (FT-IR), Taylor and Zografi analyzed the carbonyl stretching vibration of PVP in colyophilized amorphous sugar/PVP dispersions, demonstrating the formation of hydrogen bonding between the carbonyl group on PVP and hydroxyl sugar groups.^27^ The extent of these interactions was a function of the sugars chosen. Greater hydrogen bonding was noted between PVP and sucrose in comparison to trehalose, which was related to the lower *T*_g_ of sucrose (Table S1, Supporting Information). The intermolecular interactions between sugar and polymer were also studied by Shamblin et al.^26^ They analyzed differences in hydrogen bonding between sucrose with PVP and sucrose with PVPVA. PVPVA, a copolymer containing vinyl pyrrolidone and vinyl acetate at a molar ratio of 60:40, is less hygroscopic than PVP.^28,29^ A reduced degree of hydrogen bond formation was noted for the sucrose–PVPVA system compared to that of the sucrose–PVP system.

The glassy stability of these colyophilized sugar–polymer dispersions can be undermined by moisture acting as a strong plasticizer. Molecular interactions between water and the solid dispersion components can cause an increase in free volume, which allows greater local mobility resulting in a decrease in *T*_g_.^30^ The destabilizing effects of humidity on amorphous stability are highly relevant in practice due to inherent levels of residual moisture in pharmaceuticals, manufacturing environments, and components, i.e., vial stoppers. The presence of sorbed water was shown to disrupt drug–PVP intermolecular interactions.^31^ Zhang and Zografi studied the water absorption behavior of sucrose–PVP and trehalose–PVP colyophilized formulations generating moisture sorption isotherms.^32^ Their study noted that the uptake of moisture by the colyophilized formulations could be predicted by the uptake of individual components and therefore was not affected by sugar–polymer intermolecular interactions.

Studies referred to above provide a good understanding of the mechanisms by which sugar–polymer molecular interactions and thermal behavior stabilize sugars in an amorphous state within sugar–polymer amorphous dispersions. The goal of this study is to build on those earlier studies and investigate the stability of these colyophilized sugar–polymer systems across the formulation lifecycle, from the frozen solution state during the lyophilization primary drying step through to a dried state postlyophilization, and upon exposure to environment humidity during storage. The study presented also highlights how differences in sugar–polymer hydrogen bonding impact amorphous stability with respect to both temperature and humidity.

## 2 Materials and Methods

### 2.1 Materials

Sucrose and Trehalose were obtained from Pfanstiehl Inc. (USA), and polyvinylpyrrolidone (PVP K30) and poly(vinylpyrrolidone vinyl acetate) (PVPVA 64) were obtained from BASF Inc. (Germany). Vials used for lyophilization were 10 mL ISO Clear Type I Tubular glass vials, and stoppers were 20 mm finish gray silicone stoppers designed for lyophilization obtained from Adelphi Healthcare Packaging (UK).

### 2.2 Sugar–Polymer Solid Dispersion Preparation

Lyophilized solid dispersions were prepared at sugar/polymer ratios of 10:0, 8:2, 6:4, 4:6, 2:8, and 0:10. Solutions containing 10% w/v solid content were freeze-dried with a laboratory-scale freeze-dryer from VirTis AdVantage (USA). Sample aliquots of 2.5 mL were transferred into 10 mL lyophilization vials and placed on the lyophilizer shelf, which was held at 5 °C for 30 min.

Samples were frozen by cooling the shelves to −40 °C at a rate of 0.5 °C/min. After the samples were held at this temperature for 3.5 h, primary drying was started by reducing chamber pressure to 50 mTorr and continued by increasing the shelf temperature to −30 °C for 62 h. Secondary drying was initiated by increasing shelf temperature to 35 °C at a rate of 0.5 °C/min, while maintaining chamber pressure at 50 mTorr for 6 h.

Sugar–polymer solid dispersions were also prepared from molten mixtures to establish the *T*_g_ in corresponding sugar–polymer dispersions where residual moisture was minimized/eliminated by heating. These samples referred to as “melt-cooled” mixtures were prepared in situ in the differential scanning calorimetry (DSC) instrument, Q1000 TA Instruments Inc, (USA). Sugar/polymer ratios (10:0, 8:2, 6:4, 4:6, 2:8, 0:10) were mixed with a pestle and mortar. Samples of approximately 5 mg were placed in nonhermetically sealed aluminum pans to facilitate moisture escape and solid dispersions prepared by a heating and cooling cycle in the DSC instrument. Samples were heated to 200 °C and then cooled to 5 °C at a rate of 10 °C/min under dry nitrogen purge. All sugar–polymer dispersions were prepared in triplicate.

### 2.3 Characterization of Sugar–Polymer Solutions

The glass transition temperature of maximally freeze-concentrated amorphous phase (*T*_g_’) of the sugar–polymer frozen solutions was determined by DSC. Solutions of 7.5 μL were transferred to aluminum hermetically sealed pans and analyzed under a dry nitrogen purge. *T*_g_’ analysis was performed by cooling the sample to −60 °C and reheating to 20 °C at a ramp rate of 5 °C/min. The glass transition values were determined using Universal Analysis 2000 software from TA Instruments Inc. (USA). All samples were analyzed in triplicate.

### 2.4 Characterization of Sugar–Polymer Solid Dispersions

#### 2.4.1 Glass Transition Determination

The *T*_g_ of colyophilized and “melt-cooled” sugar–polymer solid dispersions were determined by DSC analysis. Lyophilized samples were analyzed in modulated mode to separate the overlapping thermal events associated with residual moisture loss and *T*_g_.^33^ In order to minimize ambient moisture uptake by samples when sampling from lyophilization vials, samples of approximately 3 mg were transferred to hermetically sealed aluminum pans working in the humidity control gloves box (relative humidity (RH) < 10%). Analysis was performed by cooling the sample to −40 °C and then ramping to 200 °C at a heating rate of 3 °C/min with an amplitude of ±1 °C and 40 s period of modulation. The *T*_g_ of “melt-cooled” samples were determined immediately postpreparation by heating samples to 200 °C at a rate of 10 °C/min under dry nitrogen purge. The measurements were conducted in triplicate for each independent sample. Universal Analysis software was used to determine the glass transition temperatures.

#### 2.4.2 Residual Moisture Determination

Residual water in the lyophilized samples was measured by Karl Fischer titration using a Moisture Meter (CA-31, Mitsubishi Chemical Analytech Co., Ltd, Japan) by external extraction using anhydrous methanol.

#### 2.4.3 FT-IR Analysis

Infrared (IR) spectral analysis was conducted using a Spectrum Two FT-IR spectrometer (PerkinElmer, UK) to detect hydrogen bond formation between sugar and polymer in lyophilized samples. Fourier Transform Infrared spectra were recorded over a wavenumber range of 400–4000 cm^–1^ in transmittance mode. Thirty-two scans were collected at a resolution of 2 cm^–1^ for each sample. The spectra were analyzed using SpectraGryph software (Germany).

#### 2.4.4 Dynamic Vapor Sorption

A dynamic vapor sorption (DVS) technique was used to measure the moisture sorption behavior of lyophilized solid dispersions and controls using a DVS Intrinsic Instrument and DVS Intrinsic Control software (Surface Measurement Systems, UK). In order to minimize variability in environmental moisture uptake when extracting samples from lyophilization vials, samples were transferred from the sealed vial to the DVS instrument in a humidity-controlled glovebox (RH < 10%). Approximately 50 mg of samples were analyzed and initially dried at 0% RH for 6 h to establish a dry mass at a constant temperature (25 ± 0.1 °C). The sample was then exposed to linear increases in relative humidity from 10 to 90% RH at a ramping rate of 10% RH/h.

## 3 Results

### 3.1 *T*_g_’ of Sugar–Polymer Solutions

**Figure 1:**
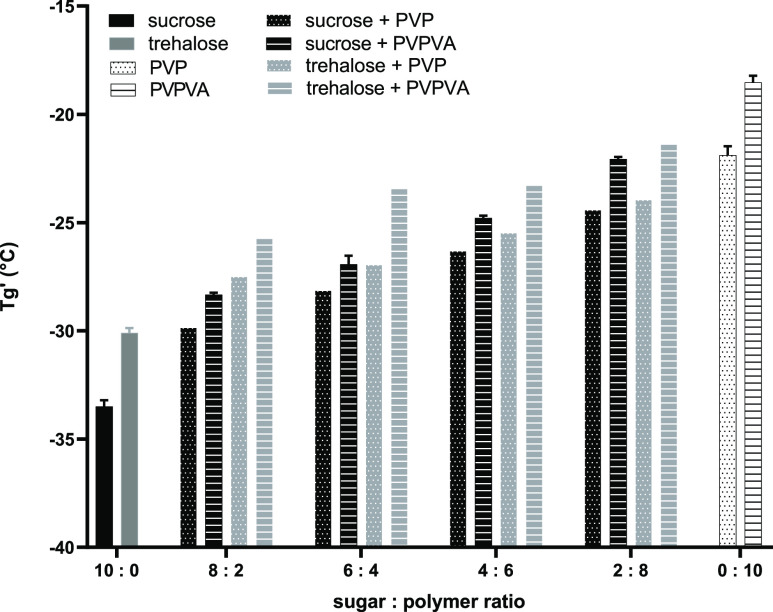
Antiplasticizing effect of polymers (PVP and PVPVA) on the *T*_g_’ of sugar (sucrose and trehalose) 10% w/v aqueous solutions. Average values shown *n* = 3, *y*-error bars indicate standard deviation.

*T*_g_’ is a critical formulation parameter to consider when designing lyophilization processes. It determines the maximal permissible product temperature during primary drying as exceeding *T*_g_’ can result in cake collapse that can further impact residual water content, amorphous sugar stability, and protein stability.^34^ The determined *T*_g_’ of the sugar–polymer solutions investigated are summarized in Figure 1.

Sucrose solutions exhibited a marginally lower *T*_g_’ than trehalose which is in agreement with previously reported values; *T*_g_’ of sucrose −33.3 ± 0.2 °C and *T*_g_’ of trehalose −29.9 ± 0.3 °C.^35^ As the *T*_g_’ is related to the plasticization of amorphous sugar by unfrozen water,^36,37^ with the reported *T*_g_ of water being −135 °C,^38^ this difference would relate to the higher *T*_g_ of trehalose compared to sucrose, detailed in Table S1 Supporting Information. In contrast, the same rationale cannot be applied to polymer only solutions, with PVP displaying a lower *T*_g_’ compared to PVPVA, despite having a higher *T*_g_ value, Table S1 Supporting Information. The lower PVP *T*_g_’ can be explained by an increase of the amount of unfrozen water in the amorphous matrix^37^ resulting in a greater plasticizing effect on PVP compared to PVPVA. For all sugar/polymer combinations, the *T*_g_’ increased with polymer addition due to their antiplasticizing effect. While higher *T*_g_’ values were measured for sugar–PVPVA compared with sugar–PVP systems, the increase in *T*_g_’ with an increase in both polymers followed a similar trend for each sugar.

### 3.2 Residual Water Content Colyophilized Systems

The residual water content was compared across all lyophilized sugar–polymer mixtures and polymer and sugar only controls (Table 1). Despite the residual water content of PVPVA alone being lower than that of PVP, this trend did not extend to colyophilized sugar and polymer systems. Factors affecting residual moisture are multifaceted including cake structure, cake resistance, and sublimation rate. The results obtained indicate that the use of a polymer with lower hygroscopicity does not necessarily result in an overall lower residual water content for lyophilized sugar–polymer systems.

**Table 1:** Water Content Values of Colyophilized Formulations as a Function of Sugar–Polymer Ratio (*n* = 3)^a^

sugar/polymer ratio	sucrose–PVP (% water)	sucrose–PVPVA (% water)	trehalose–PVP (% water)	trehalose–PVPVA (% water)
10:0	0.64 ± 0.29	0.64 ± 0.29	0.89 ± 0.10	0.89 ± 0.10
8:2	0.54 ± 0.07	0.76 ± 0.24	0.18 ± 0.07	0.44 ± 0.22
6:4	0.51 ± 0.09	1.07 ± 0.11	0.22 ± 0.02	0.33 ± 0.09
4:6	1.10 ± 0.31	1.28 ± 0.15	0.21 ± 0.05	0.39 ± 0.26
2:8	0.20 ± 0.04	0.08 ± 0.01	0.35 ± 0.01	0.31 ± 0.06
0:10	0.74 ± 0.05	0.54 ± 0.08	0.74 ± 0.05	0.54 ± 0.08

a Water content is expressed as a percentage of the theoretical lyophilized cake mass.

### 3.3 Glass Transition Temperature of Colyophilized Systems

**Figure 2:**
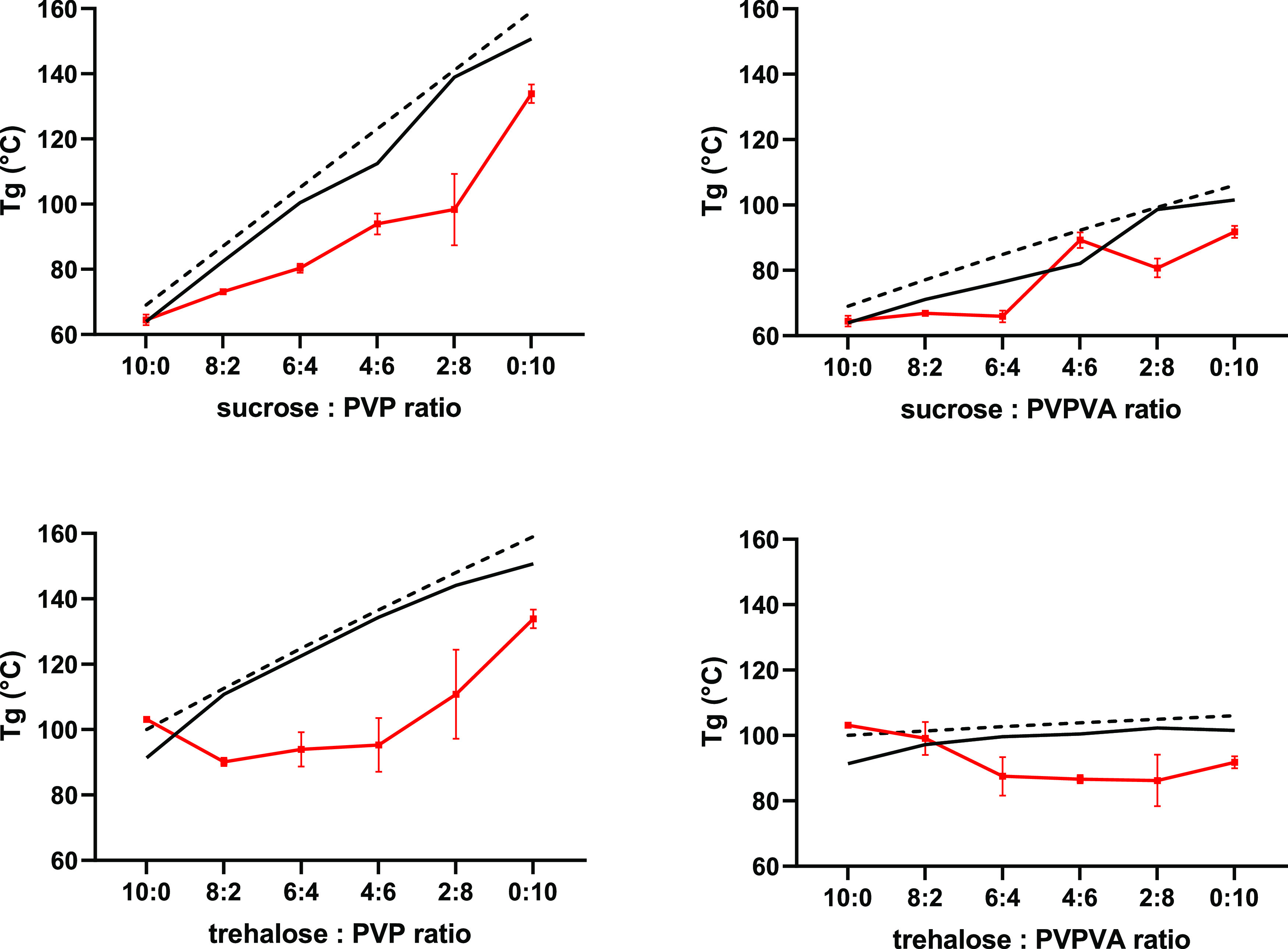
Glass *T*_g_ of sucrose–PVP, sucrose–PVPVA, trehalose–PVP, and trehalose–PVPVA lyophilized mixtures in red as a function of sugar/polymer ratio. *n* = 3, average values shown; *y*-error bars indicate standard deviation. The dashed line represents the *T*_g_ values predicted by the Gordon–Taylor equation for binary mixtures (eq 1), and the solid line represents the *T*_g_ values predicted by the Gordon–Taylor equation for ternary mixtures (eq 2).

The *T*_g_ of the colyophilized sugar–polymer systems are shown in Figure 2. Lyophilized polymer and sugar only systems are included as controls. The experimentally determined values were compared with theoretical *T*_g_ values predicted using the Gordon–Taylor equation considering a binary system sugar and polymer only and a ternary system considering residual water as a third component, as shown in eqs 1 and 2.
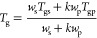
1
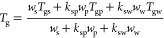
2where *w*_s_, *w*_p_, and *w*_w_ are the mass fractions of each component and *T*_gs_, *T*_gp_, and *T*_gw_ are the glass transition temperatures of the individual components. The constants *K*_sp_ and *k*_sw_, which are a measure of interaction between the components, can be calculated using the Simha–Boyer rule; *k*_sp_ = ρ_s_*T*_gs_/ρ_p_*T*_gp_ and *k*_sw_ = ρ_s_*T*_gs_/ρ_w_*T*_gw_, where ρ is the density of each component (sucrose: 1.59 g/cm^3^,^39^, trehalose: 1.58 g/cm^3^,^40^, PVP: 1.25 g/cm^3^,^41^, PVPVA: 1.27 g/cm^3^,^42^, water: 1 g/cm^3^,^43^). The measured residual water content values in Table 1 were employed, and *T*_g_ values of each component used in these calculations are detailed in Table S1 Supporting Information.

For the colyophilized sugar–PVP systems, the experimentally determined *T*_g_ values deviated negatively from the *T*_g_ values predicted by the Gordon–Taylor binary and ternary equations. In contrast, the negative deviation between the measured and predicted *T*_g_ values for the sugar–PVPVA systems was reduced and was not observed for all samples. Negative deviation from the predicted *T*_g_ values can be attributed to sugar–polymer molecular interactions. Negative deviation from the ideal behavior described by the Gordon–Taylor equation indicates that sugar–PVP interactions have reduced strength compared to sugar–sugar interactions. In contrast, the negative deviation between the measured and predicted *T*_g_ values for the sugar–PVPVA systems indicates greater similarity between sugar–PVPVA and sugar–sugar interactions.^39−42^

**Figure 3:**
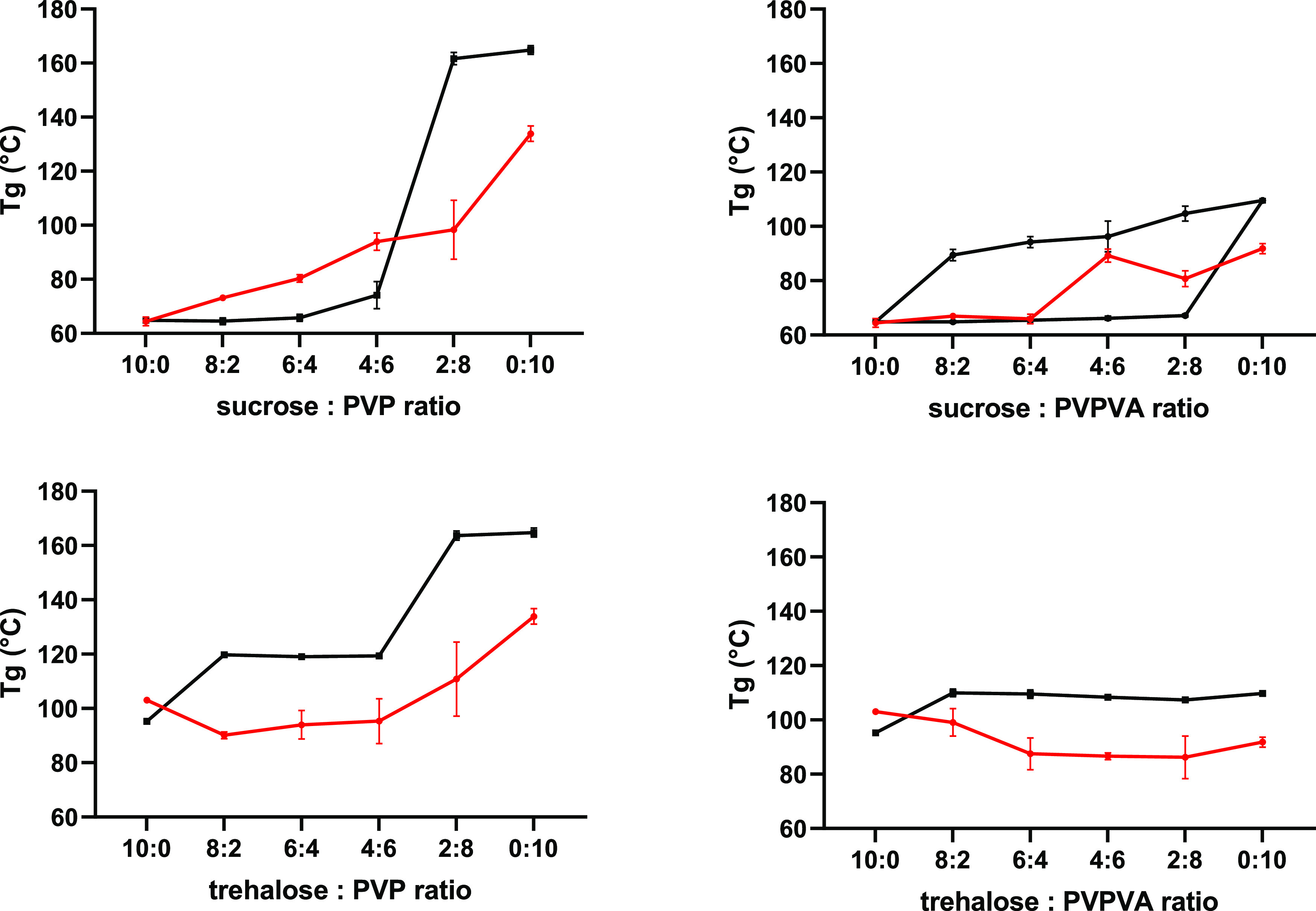
Glass *T*_g_ of sucrose–PVP, sucrose–PVPVA, trehalose–PVP, and trehalose–PVPVA lyophilized mixtures in red and “melt-cooled” mixtures in black as a function composition. *n* = 3, average values shown, *y*-error bars indicate standard deviation.

To investigate the effect of the lyophilization process on sugar–polymer molecular interactions and *T*_g_, sugar–polymer solid dispersions were prepared from molten mixtures. These systems were referred to as “melt-cooled” mixtures, and the *T*_g_ values of these mixtures were compared with the *T*_g_ values of the corresponding colyophilized systems, Figure 3.

All “melt-cooled” mixtures displayed a single *T*_g_ value indicating the detection of a single amorphous phase except the sucrose–PVPVA systems, which exhibited two *T*_g_ values similar to the individual components, indicating poor mixing in the molten state during preparation. *T*_g_ differences between the “melt-cooled” and colyophilized systems can be attributed to two possible factors. Heating during the “melt-cooled” method can eliminate water content while the colyophilized samples contain residual moisture (Table 1). The second factor for consideration is the degree of sugar–polymer mixing during preparation, facilitating intermolecular interactions between sugar and polymer. Increased mixing in the colyophilized systems is expected as they are prepared from sugar and polymer molecularly dispersed in the solution state.

Notably, sucrose–PVP colyophilized samples displayed a higher *T*_g_ than the “melt-cooled” samples up to 60% PVP, indicating the increased antiplasticization by the polymer in these systems due to increased mixing offsetting the plasticizing effect of residual moisture. The plasticizing effect of water is evident for the 80% PVP sample and the PVP-only control. In contrast, the trehalose–polymer colyophilized samples displayed lower *T*_g_ values than the corresponding “melt-cooled” samples. The exact cause for these differences between sucrose and trehalose systems is difficult to conclude as a combination of factors may be involved; the residual water plasticizing effect coupled with efficient sugar–polymer mixing in the “melt-cooled” samples or reduced hydrogen bonding between trehalose and the carbonyl polymer groups are previously reported.^24^

### 3.4 FT-IR Analysis of Colyophilized Systems

**Figure 4:**
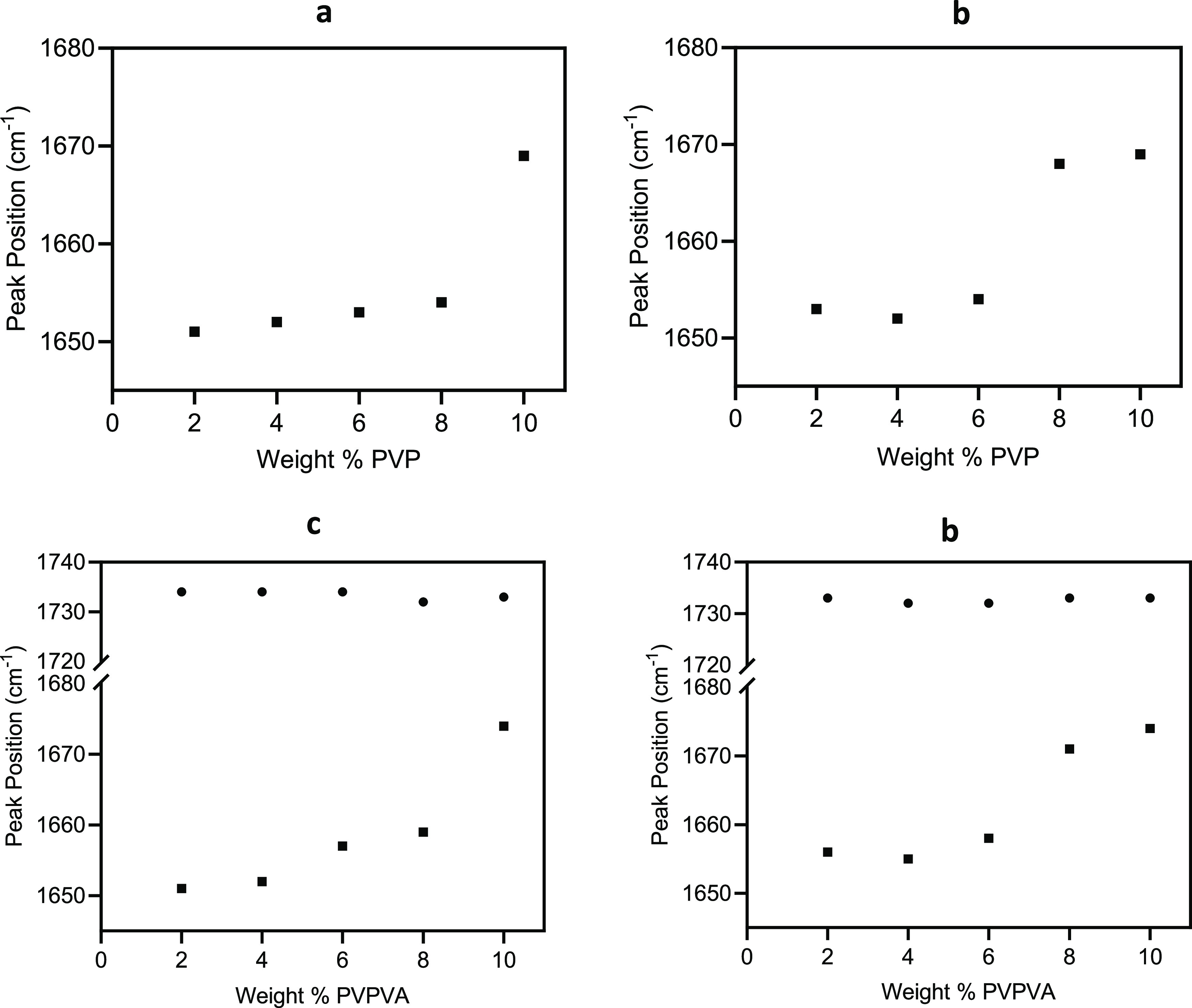
FT-IR peak position of the carbonyl stretch of the vinyl pyrrolidone moieties for (a) sucrose–PVP mixtures and (b) trehalose–PVP mixtures. Peak position of the C=O stretching region of the vinyl pyrrolidone and vinyl acetate moieties in (c) sucrose–PVPVA and (d) trehalose–PVPVA mixtures.

The FT-IR spectrum peak position of the carbonyl stretch of the vinyl pyrrolidone ring of both polymers, which is an indicator of hydrogen bonding with sugars in the colyophilized systems, is shown in Figure 4. The results obtained agree with the findings of Taylor and Zografi.^27^ It was observed that by increasing the percentage of sugar in the system, the carbonyl peak shifted to lower wavenumbers from 1669 to 1652 cm^–1^. This shift was observed for all sucrose–PVP systems studied and for trehalose–PVP systems up to 60% PVP. At 80% PVP, the notable shift was not evident, indicating hydrogen bonding in trehalose–PVP was not detected. Sugar–PVP hydrogen bonds fix the sugar molecules around PVP molecules, and this prevents sugar molecules from orienting themselves to form sugar–sugar hydrogen bonds.^43^

For sugar–PVPVA systems, two C=O peaks were observed at 1674 and 1733 cm^–1^, attributed to the carbonyl groups of vinyl pyrrolidone and vinyl acetate, respectively. In agreement with what was observed for the sugar–PVP systems, the peak position of PVP carbonyl shifts to lower wavenumbers as the percentage of sugar increases following the same trend. However, the 1669 cm^–1^ shift to the lowest wavenumber was observed only for systems with higher sugar/polymer ratios. No noticeable shift was observed for the C=O of the vinyl acetate peak, which indicates weak sugar–polymer interaction. The carbonyl group of the pyrrolidone ring is a stronger base than the carbonyl group which is contained in the vinyl acetate portion.^44^ Similar to the trehalose–PVP system, a notable shift was not evident at the 2:8 trehaolse/PVPVA ratio.

### 3.5 Water Sorption Analysis of Colyophilized Systems

**Figure 5:**
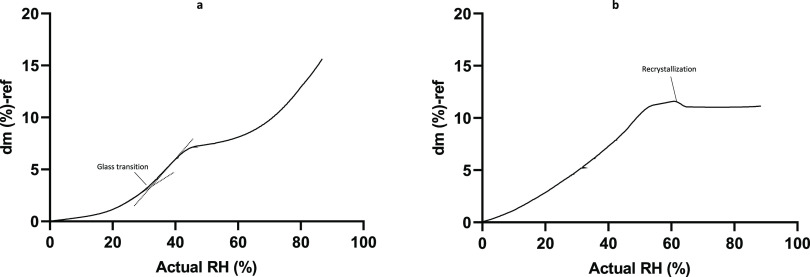
Uptake of moisture during humidity ramping (10% RH/h) at 25 °C for (a) lyophilized sucrose and (b) lyophilized trehalose sample, with onset glass transition humidity (RH_g_) and onset crystallization humidity (RH_c_) behavior highlighted.

The colyophilized sugar–polymer systems and sugar and polymer only controls were exposed to increasing relative humidities at 25 °C ramping from 0 to 90% RH at 10% RH per hour. For each system, an onset glass transition humidity (RH_g_) was determined at the transition point where a shift in sorption characteristics occurs due to increased bulk absorption as material mobility increases above its glass transition (Figure 5).^45−48^ This shift in water sorption rate at the RH_g_ is indicated by the intersecting tangents for the lyophilized sucrose system shown in Figure 5a.

At low RH values, water uptake is limited to available discrete hydrogen binding sites and accessible interstitial spaces of the dense, rigid, glassy material.^49^ However, when the material undergoes glass transition, the molecular mobility increases and moisture uptake increases allowing bulk water absorption by the increasingly flexible and rubbery amorphous material.^50,51^ The detection of transitions from amorphous to crystalline states, the onset crystallization humidity (RH_c_), can also be determined as illustrated for the lyophilized trehalose sample, Figure 5b. As the humidity levels increase, the water uptake rate increases and RH_c_ is determined as the point when a characteristic loss in mass occurs (negative d*m*/d*t*) due to dihydrate crystal formation.^48,52^ For trehalose, the final mass stabilizes at 10.8% water uptake, which agrees with the stoichiometric water content of trehalose dihydrate, with water composing 10.5% of the anhydrous trehalose molecular weight.

**Figure 6:**
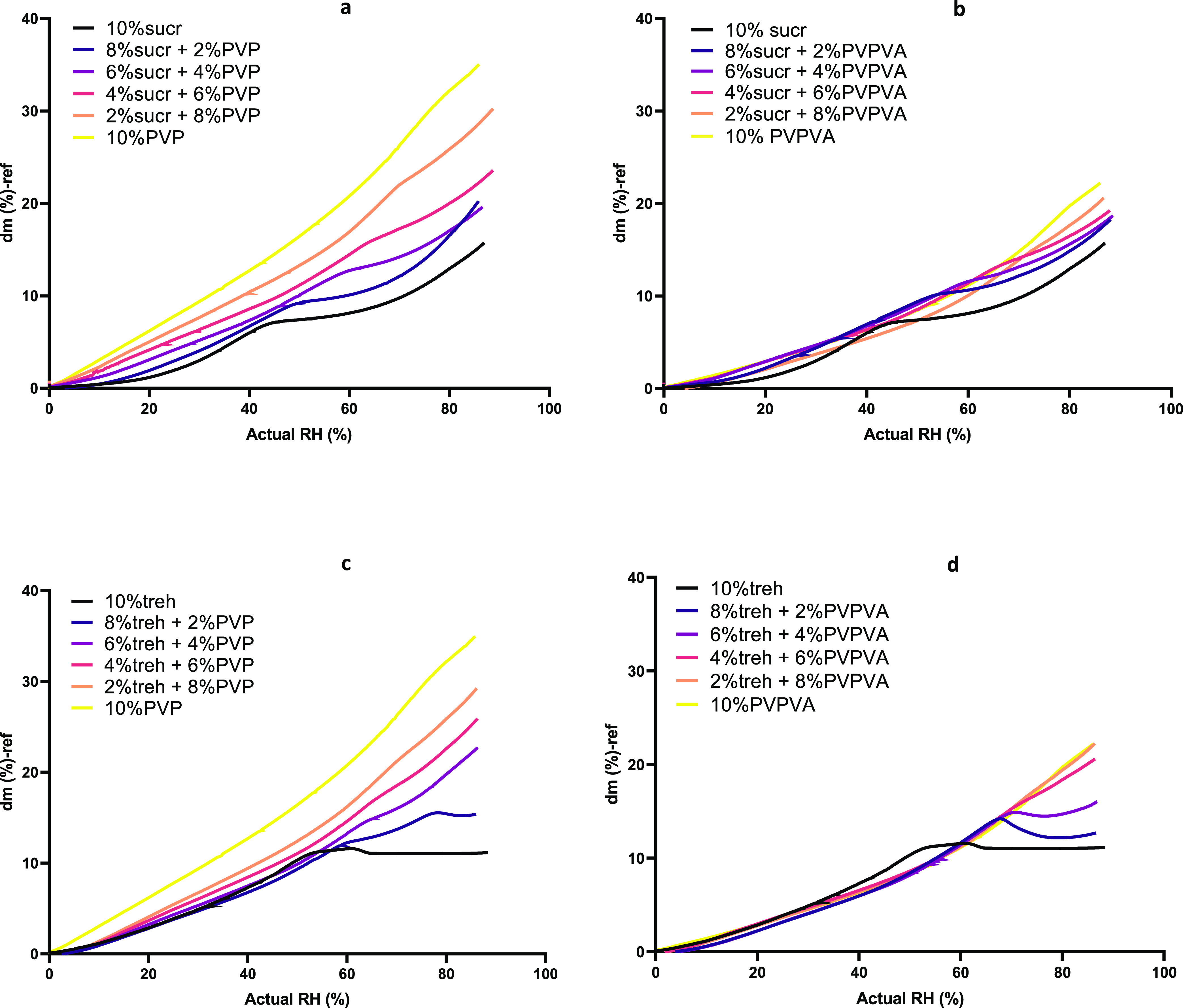
Moisture uptake profile during humidity ramping (10% RH/h) at 25 °C for colyophilized samples of (a) sucrose–PVP, (b) sucrose–PVPVA, (c) trehalose–PVP, and (d) trehalose–PVPVA.

The moisture sorption profiles for colyophilized sugar/polymer systems exposed to the same ramping humidity profile are shown in Figure 6, and the determined RH_c_ and RH_c_ values are listed in Table 2.

**Table 2:** Onset Glass Transition Humidity (RH_g_) and Onset Crystallization Humidity (RH_c_) Determined from Humidity Ramping Experiment (10% RH/h) at 25 °C

	sucrose–PVP		sucrose–PVPVA		trehalose–PVP		trehalose–PVPVA	
sugar/polymer ratio	RH_g_ (°C)	RH_c_ (°C)	RH_g_ (°C)	RH_c_ (°C)	RH_g_ (°C)	RH_c_ (°C)	RH_g_ (°C)	RH_c_ (°C)
10:0	25		25			64		64
8:2	28		30		43	78		68
6:4	45		41		52		52	73
4:6	49		50		56		57	
2:8	60		59		58		59	
0:10	65		64		65		64	

For all colyophilized sugar–polymer systems, as the polymer ratio increased, the onset glass transition humidity values increased. It was not possible to accurately determine the values for lyophilized trehalose systems and trehalose–PVPVA systems with a ratio of 8:2 trehalose/PVPVA and these were omitted from Table 2. It is clear that increasing the percentage of polymer, regardless of whether it is PVP or PVPVA, increased the humidity level at which the glass transition was induced. However, differences were noted between the polymers in terms of the amount of water uptake, Figure 6. The water uptake for PVP systems with both sugars increases rapidly with an increase in humidity. In the case of PVPVA systems with both sugars, the water uptake rate is slower and similar for all colyophilized sugar–PVPVA systems. These differences are expected due to the reduced hygroscopicity of PVPVA compared to PVP.^28,29^

This humidity ramping analysis also highlighted differences in the relative amorphous stability of the sugars alone and when colyophilized with polymers. For lyophilized trehalose, crystallization occurred at 63% RH. However, when colyophilized with polymer trehalose, recrystallization occurred at higher relative humidities or was inhibited. Inhibition of trehalose recrystallization occurred with a lower percentage of PVP compared to PVPVA, indicating that PVP provided greater amorphous stability, despite the greater moisture uptake by the trehalose–PVP systems. This stabilization effect may be attributed to the increased hydrogen bonding between trehalose and PVP compared to trehalose–PVPVA.

## 4 Discussion

The study presented shows how colyophilization of stabilizing sugars with high *T*_g_ polymers (PVP and PVPVA) can stabilize the sugars investigated (sucrose and trehalose) in an amorphous state during production and storage. The results highlight how the mechanism of stabilization is dependent on the polymer and sugar combination and ratio and differs for different lyophilization processing stages and storage stresses. The findings of the study have implications that can be applied to commercial biopharmaceutical products and process design. For example, despite PVPVA having a lower reported *T*_g_ value than PVP,^53−55^ 10% PVPVA solution exhibited a higher *T*_g_’ value than the corresponding PVP solution. PVPVA also exhibited a greater antiplasticization effect on frozen sugar solution *T*_g_’ compared to PVP, Figure 1. These differences were attributed to PVPVA’s weaker hydrogen bonding capacity leading to reduced interaction with water and decreased plasticizing unfrozen water. The higher *T*_g_’ of PVPVA compared to PVP would enable the lyophilization primary drying step to be conducted at higher temperatures, reducing lyophilization cycle times, and avoiding cake collapse and product destabilization.^35^

It could be anticipated that the *T*_g_ values for the colyophilized sugar–PVPVA systems would contain lower residual moisture than PVP systems when subjected to the same drying process due to PVPVA’s lower hygroscopicity. The results showed no clear reduction in residual water content for colyophilized systems containing PVPVA compared to PVP. While the colyophilized system’s affinity for moisture is one factor that can affect lyophilized cake residual moisture, residual moisture is also highly dependent on cake porosity and resistance, which in turn can be influenced by ice nucleation during freezing.^56^

Despite the reported *T*_g_ of PVP being higher than PVPVA, this did not translate to substantially higher *T*_g_ values for colyophilized sugar–PVP systems compared to the corresponding PVPVA systems. The relative increased hygroscopicity of PVP and its greater capacity for hydrogen bonding with sugars compared to PVPVA resulted in a plasticizing effect. Interestingly, while PVP hygroscopicity did result in a higher uptake of moisture in the humidity ramping study, it inhibited trehalose recrystallization at lower ratios than PVPVA, which was attributed to its enhanced hydrogen bonding capabilities. Compared to trehalose, sucrose forms stronger hydrogen bonds with the pyrrolidone carbonyl group in PVP and PVPVA, which contributed to the stabilization of the amorphous state at elevated humidities.

Based on the results obtained, it could be concluded that both PVP and PVPVA can produce colyophilized amorphous systems with sugars that are stable with respect to elevated temperatures up to 80 °C in the absence of humidity, Figure 3. This finding is relevant for lyophilized biopharmaceutical parenteral products that are sealed under a vacuum or with backfilling with dry nitrogen following vial filling. For protein therapeutics, a high ratio of sugar to polymer would be critical as the sugar component is the stabilizing agent interacting with the therapeutic molecule. The polymer in the absence of the sugar fails to inhibit protein unfolding during lyophilization and the rate of degradation during storage is significantly increased for unfolded proteins without the inclusion of a sugar stabilizer.^3,57^

Increasingly, there is interest in protein and vaccine stabilization in amorphous sugar matrices for nonparenteral delivery such as intradermal microneedles.^58,59^ Unlike parenteral formulations, these solid formulations are exposed to environmental humidity during manufacture and between the opening of packaging and administration. Therefore, mechanisms of amorphous state stabilization with respect to the combined factors of temperature and humidity are highly relevant. Based on the findings of this study, a sucrose–polymer dispersion would provide better stability than a trehalose–polymer dispersion which was shown to have a higher propensity to recrystallize. In addition to amorphous stability, which was the focus of this study, for dissolvable microneedle formulations, the impact of moisture uptake on needle mechanical strength and skin penetration would also need to be considered.^60,61^

Finally, a perceived limitation of the work presented could be that it does not include a therapeutic protein or biopharmaceutical active ingredient in the systems studied. The variable physicochemical properties of biopharmaceutical active ingredients, considering peptides, proteins, antibody-drug conjugants, viral vectors, and nucleic acids, can lead to complex and diverse interactions with excipients during processing and storage. The advantage of conducting the studies presented in the absence of a therapeutic agent provides a fundamental understanding of the solid-state behavior of these pharmaceutical excipient systems which can inform formulation design for a range of biopharmaceutical actives.

## 5 Conclusions

The study presented demonstrates how differences in PVP and PVPVA hygroscopicity and hydrogen bonding with sucrose and trehalose influence the stability of colyophilized amorphous dispersions. Sugar–PVPVA frozen solutions exhibited a higher *T*_g_’ which is desirable for stability during lyophilization primary drying. Despite PVP having a higher *T*_g_ than PVPVA, the sugar–PVP colyophilized systems were more susceptible to plasticizing effects compared to their sugar–PVPVA counterparts. Sucrose systems exhibited greater amorphous stability when exposed to elevated humidity compared to that of trehalose. Despite having greater water uptake, greater stability was observed for trehalose–PVP systems compared to trehalose–PVPVA. Trehalose–polymer hydrogen bond capacity was shown to be an important stabilizing factor when colyophilized systems are exposed to elevated humidity.

## Supporting Information Available

Glass transition temperature values (*T*_g_) for single components (PDF)
