# Supplementary material for: Colyophilized Sugar–Polymer Dispersions for Enhanced Processing and Storage Stability
Source: Mol Pharm. 2024 May 17;21(6):3017–26. doi: 10.1021/acs.molpharmaceut.4c00187 (PMC11151204; doi:10.1021/acs.molpharmaceut.4c00187)
Supplement: Supplementary file 1 — mp4c00187_si_001.pdf [file mp4c00187_si_001.pdf]

Co-lyophilized sugar-polymer dispersions for enhanced processing and storage stability

Claudia Giannachi[1,2], *Evin Allen*[2], *Gráinne Egan*[2], *Sonja Vucen*[1,2], *Abina Crean*[1,2]

[1] SSPC, the SFI Research Centre for Pharmaceuticals, School of Pharmacy, University College Cork, T12 YT20, Ireland

[2] School of Pharmacy, University College Cork, T12 YT20, Ireland

Supporting Information for Publication

Table S1: Glass transition temperature, Tg, for single components

Material	Onset Tg (°K)	Reference
sucrose	342	[S1]
trehalose	373	[S2]
PVP K30	432	[S3]
PVPVA 64	379	[S4]
water	138	[S5]

References

S1. Katkov, I. I.; Levine, F. Prediction of the Glass Transition Temperature of Water Solutions: Comparison of Different Models. Cryobiology 2004, 49, 62–82. https://doi.org/10.1016/j.cryobiol.2004.05.004.

S2. Chen, T.; Fowler, A.; Toner, M. Literature Review: Supplemented Phase Diagram of the Trehalose-Water Binary Mixture. Cryobiology 2000, 40, 277–282. https://doi.org/10.1006/cryo.2000.2244.

S3. Phadke, C.; Sharma, J.; Sharma, K.; Bansal, A. K. Effect of Variability of Physical Properties of Povidone K30 on Crystallization and Drug–Polymer Miscibility of Celecoxib–Povidone K30 Amorphous Solid Dispersions. Mol Pharm 2019, 16 (10), 4139–4148. https://doi.org/10.1021/acs.molpharmaceut.9b00452.

S4. Goddeeris, C.; Willems. T.; Van den Mooter, G. Formulation of Fast Disintegrating Tablets of Ternary Solid Dispersions Consisting of TPGS 1000 and HPMC 2910 or PVPVA 64 to Improve the Dissolution of the Anti-HIV Drug UC 781. European Journal of Pharmaceutical Sciences 2008, 34, 293–302. https://doi.org/10.1016/j.ejps.2008.05.005.

S5. Johari, G. P.; Hallbrucker, A.; Mayer, E. The Glass–Liquid Transition of Hyperquenched Water. Nature 1987, 330 (6148), 552–553. https://doi.org/10.1038/330552a0.
